# P27 A scoping review of interventions to improve blood culture sampling in acute care settings.

**DOI:** 10.1093/jacamr/dlaf118.034

**Published:** 2025-07-14

**Authors:** Muuna Abdi, Deborah Bamber, Carolyn Tarrant

**Affiliations:** School of Medicine, University of Leicester, Leicester, UK, LE1 7RH; Department of Population Health Sciences, University of Leicester, Leicester, UK, LE1 7RH; Department of Population Health Sciences, University of Leicester, Leicester, UK, LE1 7RH

## Abstract

**Background:**

Blood cultures (BCs) are an essential diagnostic investigation in patients presenting acutely with suspected severe infection and sepsis. Up to half of emergency admissions do not have BCs taken when antibiotics are started as recommended by best practice guidelines. Additionally, when they are taken, problems arise from low volume filling, single set collection and user contamination. Optimizing the BC sampling pathway has several benefits, notably decreasing inappropriate antibiotic therapy to improve antimicrobial stewardship. Although much progress has been made in the early identification and management of sepsis, significant potential for improving BC sampling practices for patients with sepsis and severe infection remains.

**Objectives:**

The purpose of this scoping review is to identify evidence on the types of interventions applied to improve BC sampling practices in higher economically developed countries and subsequently evaluate these strategies and their implementation.

**Methods:**

This scoping review identifies interventions to improve BC sampling for adults presenting to acute care settings across higher economically developed countries. Database searches of Medline, CINAHL, PubMed and BMJ Open Quality between January 2015 and January 2025 were conducted. Reference and citation lists were also searched. Findings were mapped to the Behaviour Change Wheel (BCW) framework to identify intervention types; in doing so, the most common intervention types could be identified and opportunities for new intervention approaches highlighted. Factors impacting on intervention implementation were also evaluated.

**Results:**

Database searches returned 3744 transcripts and after screening, 21 papers were eligible for inclusion with two further studies identified through reference lists. Of the 23 papers analysed, 6 intervention types were identified. The most common interventions were ‘Environmental Restructuring’ (33%), ‘Education and Training’ (29%) and ‘Enablement’ (24%). ‘Environmental Restructuring’ was the most frequently implemented intervention type, with common examples including visual cues and prompts such as posters and sepsis alerts (*N*=14) sepsis algorithms and screening tools (*N*=10), and novel protocol implementation (*N*=3). These interventions modified the physical environment and social context to reduce barriers to optimal BC sampling. Audit and feedback were the most common examples of ‘Enablement’ within the studies. No studies included ‘Coercion’ or ‘Restriction’ as interventions. Common difficulties in ensuring sustainable implementation of interventions related to rapid staff turnover and a lack of resources.

**Conclusions:**

This scoping review provides detail around existing interventions to improve BC sampling in acute care settings and emphasizes the challenges of implementation. Furthermore, this review identifies a gap in UK-based studies around interventions to improve BC sampling. Further research is needed for the development of sustainable, evidence-based interventions to optimize BC sampling practices within the UK context.

